# Congenital Diaphragmatic hernia – a review

**DOI:** 10.1186/s40748-017-0045-1

**Published:** 2017-03-11

**Authors:** Praveen Kumar Chandrasekharan, Munmun Rawat, Rajeshwari Madappa, David H. Rothstein, Satyan Lakshminrusimha

**Affiliations:** 1 Department of Pediatrics, Women and Children’s Hospital of Buffalo, Buffalo, NY USA; 2 SIGMA Hospital, Mysore, India; 3 Department of Pediatric Surgery, Women and Children’s Hospital of Buffalo, Buffalo, NY USA

**Keywords:** Lung Hypoplasia, Pulmonary Hypertension, Extracorporeal membrane oxygenation

## Abstract

Congenital Diaphragmatic hernia (CDH) is a condition characterized by a defect in the diaphragm leading to protrusion of abdominal contents into the thoracic cavity interfering with normal development of the lungs. The defect may range from a small aperture in the posterior muscle rim to complete absence of diaphragm.

The pathophysiology of CDH is a combination of lung hypoplasia and immaturity associated with persistent pulmonary hypertension of newborn (PPHN) and cardiac dysfunction. Prenatal assessment of lung to head ratio (LHR) and position of the liver by ultrasound are used to diagnose and predict outcomes. Delivery of infants with CDH is recommended close to term gestation. Immediate management at birth includes bowel decompression, avoidance of mask ventilation and endotracheal tube placement if required. The main focus of management includes gentle ventilation, hemodynamic monitoring and treatment of pulmonary hypertension followed by surgery. Although inhaled nitric oxide is not approved by FDA for the treatment of PPHN induced by CDH, it is commonly used.

Extracorporeal membrane oxygenation (ECMO) is typically considered after failure of conventional medical management for infants ≥ 34 weeks’ gestation or with weight >2 kg with CDH and no associated major lethal anomalies. Multiple factors such as prematurity, associated abnormalities, severity of PPHN, type of repair and need for ECMO can affect the survival of an infant with CDH. With advances in the management of CDH, the overall survival has improved and has been reported to be 70-90% in non-ECMO infants and up to 50% in infants who undergo ECMO.

## Background

Congenital Diaphragmatic hernia (CDH) is characterized by a defect in the diaphragm leading to the protrusion of abdominal contents into the thoracic cavity affecting the normal development of the lungs. The condition may present as an isolated lesion or as part of a syndrome. The incidence of CDH based on the available literature ranges from approximately 0.8 - 5/10,000 births and varies across the population [1–4]. There is slightly higher male predominance and a lower risk of isolated CDH reported among African-Americans [3, 5]. In spite of advances made in the medical and surgical management of CDH, the mortality and morbidity remain high [6–8]. CDH infants also have a prolonged length of stay in the hospital requiring multi-disciplinary approach for their management and follow-up after hospital discharge.

## Review

### Etiology

The etiology of CDH largely remains unclear and currently is thought to be multifactorial. The majority of the cases have an isolated diaphragmatic defect presenting with pulmonary hypoplasia and persistent pulmonary hypertension of newborn (PPHN). CDH can be associated with cardiac, gastrointestinal, genitourinary anomalies or with chromosomal aneuploidy such as trisomies. Multiple genetic factors along with environmental exposures and nutritional deficiencies have been proposed to be the possible etiologies for CDH [9–11]. Studies in rodent models have pointed towards a disturbance in Vitamin A pathway [12]. Nitrofen, a herbicide, when administered to pregnant rodents, results in CDH in the majority of offspring [13, 14]. Similar effects were seen in WT1 and COUP-TFII mutant mouse models. Studies in neonates with CDH have shown low retinol and retinol-binding protein levels from cord blood samples [9, 15].

### Pathology

**Fig. 1:**
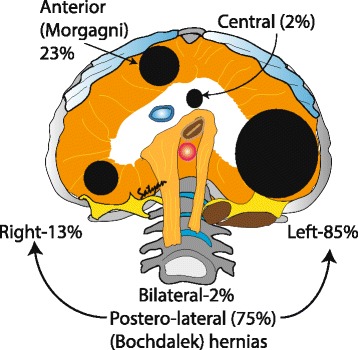
Classification of CDH based on location of the diaphragmatic hernias: Most common type of hernias are the posterior lateral hernias (70–75%) also known as Bochdalek hernias, with majority occurring on the left side (85%) and less frequently on the right side (13%) or bilateral (2%). Other types of hernias are the anterior defects or Morgagni hernias (23–28%) followed by the rare central hernias (2–7%). (*Copyright Satyan Lakshminrusimha)*

Location (Fig. 1): Postero-lateral hernias also known as Bochdalek hernias are the most common type (70–75%) with the majority occurring on the left side (85%) and less frequently on the right side (13%) or bilateral (2%). Anterior defects or Morgagni hernias (23–28%) and central hernias (2–7%) are the other types [16, 17].

**Fig. 2:**
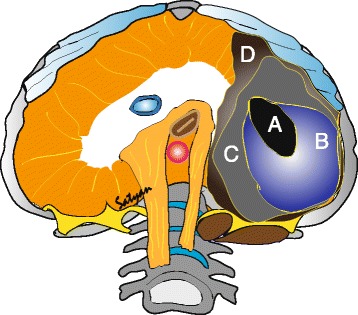
Size of the defect – The size of the defect may vary between small (A) to diaphragmatic agenesis (D). Defects B & C are considered moderate to large (Tsao et al. 2008). (*Copyright Satyan Lakshminrusimha)*

Size (Fig. 2): The diaphragm begins to develop at approximately 4 weeks of gestation and is fully formed by 12 weeks [18]. The defect may range from a small opening of the posterior muscle rim to complete absence of diaphragm.

The embryologic basis of CDH remains controversial. It was thought initially that the defect happened secondary to failure of different parts of the diaphragm to fuse resulting in a patent pleuroperitoneal canal [19, 20]. Rat models have shown a defect in the primordial diaphragm called the pleuroperitoneal fold [16]. This, in turn, allows the gut to enter the thoracic cavity when it returns from the extraembryonic coelom of the umbilicus. Another speculation is that lung hypoplasia may be the primary causal factor in the pathophysiology of diaphragmatic hernia [21]. If the development of lung bud is disturbed, there is an impaired development of a post hepatic mesenchymal plate (PHMP) that is closely related to the development of lung, resulting in a defective diaphragm [21]. Evidence from electron microscopy in a rat model [22] of CDH further supports the fact that when the development of the PHMP is impaired, a diaphragmatic defect occurs.

A weakness in the diaphragm can cause diaphragmatic eventration and may be mistaken for a diaphragmatic hernia. Diaphragmatic eventration is more common on the right side and is not associated with severe lung hypoplasia. While a complete absence of the diaphragm may occur resulting in diaphragmatic agenesis and severe lung hypoplasia. Irrespective of the basis, a defect in the diaphragm causes the abdominal viscera to herniate into the thoracic cavity resulting in abnormal lung development. The defect also leads to abnormal fetal breathing movements resulting in the void of stretch-induced lung maturation [16]. Thus the major underlying pathophysiology of CDH appears to be a combination of lung immaturity and hypoplasia that leads to PPHN. This may be further aggravated by left ventricular underdevelopment and right ventricular hypertrophy resulting in ventricular dysfunction [23–26].

#### Lung hypoplasia/immaturity

**Fig. 3:**
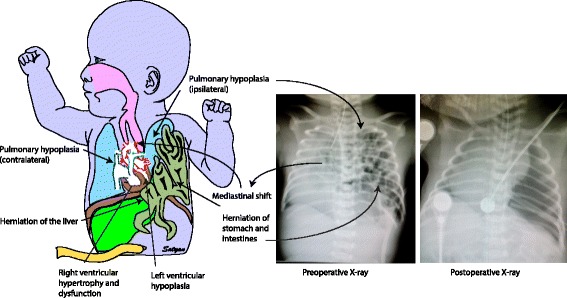
Anatomical and radiological features of CDH – A defect in the diaphragm causes the abdominal viscera to herniate into the thoracic cavity. Left sided hernias are common (85%) which results in herniation of both small and large intestines along with solid intra-abdominal organs. Herniation of viscera into the thoracic cavity results in abnormal lung development on the ipsilateral side with variable effect on the contralateral side. The effect of abnormal lung development on the contralateral side depends on the extent of herniation and the effect on mediastinal shift. Pulmonary hypoplasia results in abnormal pulmonary vasculature resulting in persistent pulmonary hypertension leading to right ventricular dysfunction. This is more pronounced after transitioning from fetal circulation. Left ventricular dysfunction can be secondary to direct compression in left sided hernia and secondary to low ventricular volumes in right sided hernias. Pre-operative chest and abdomen x-ray shows the air and fluid filled loops of bowels on the left side of the thorax with the endotracheal tube above the thoracic vertebra level 4 pushed towards the right side signifying mediastinal shift. (*Copyright Satyan Lakshminrusimha)*

**Fig. 4:**
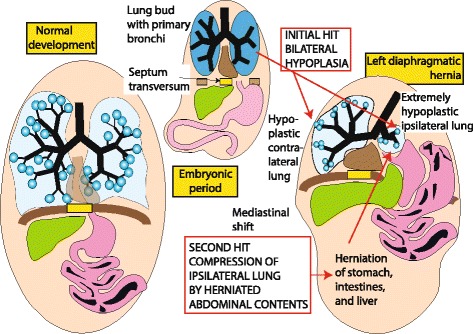
Two-hit hypothesis for CDH (*Copyright Satyan Lakshminrusimha)*

Lung hypoplasia occurs on the ipsilateral side of herniation, with the contralateral side being affected to a variable extent (Fig. 3). Hypoplasia was initially thought to be secondary to physical compression of the lung by abdominal contents arresting lung development. Recently, a two-hit hypothesis has been proposed based on rat model explaining the lung injury in CDH [27] (Fig. 4). According to this hypothesis, the initial insult occurs during the stages of organogenesis resulting in bilateral hypoplasia, followed by compression of the ipsilateral lung secondary to the herniation of the abdominal viscera at later stages. This theory explains the variability of lung hypoplasia on the contralateral side. The interference results in decreased branching of the bronchioles and pulmonary vessels leading to acinar hypoplasia [28, 29]. The terminal bronchioles are decreased with thickening of alveolar septa. The lung is relatively immature [28] and hypoplasia of pulmonary vasculature leads to PPHN.

#### Pulmonary hypertension in CDH

In CDH, the total pulmonary vascular bed is reduced with decreased number of vessels per unit of lung. In addition, pulmonary vascular remodeling with medial hyperplasia and peripheral extension of the muscle layer into small arterioles is evident [30–32]. The paucity of pulmonary vasculature and remodeling of the vessels contribute to the ‘fixed’ or irreversible component of PPHN in CDH [33, 34]. Altered vasoreactivity possibly due to an imbalance of autonomic innervation (increased sympathetic and decreased parasympathetic) [35], and/or impaired endothelium-dependent relaxation of pulmonary arteries [36, 37] or an imbalance between vasoconstrictor and vasodilator mediators may contribute to the reversible component of PPHN [35, 38]. Following birth, a combination of pulmonary arterial hypertension, right ventricular hypertrophy and/or failure, and left ventricular hypoplasia with pulmonary venous hypertension results in severe PPHN unresponsive to conventional management [39].

#### Ventricular dysfunction

**Fig. 5:**
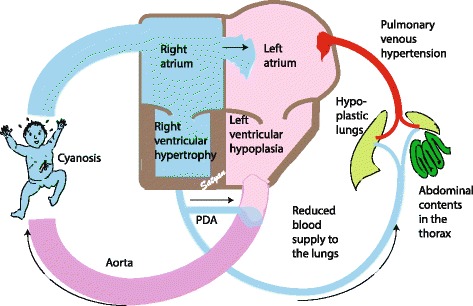
Cardiovascular effects of CDH – Hypoplastic lungs secondary to herniation of abdominal viscera leads to concomitant hypoplasia of the pulmonary vessels. This results in reduced blood supply to the hypoplastic alveolar-capillary unit. Once the infant transitions from fetal circulation, this effect is more pronounced resulting in pulmonary hypertension which leads to right ventricular dysfunction. Secondary to pulmonary hypertension, there is shunting of blood from right to left across the patent foramen ovale and the patent ductus arteriosus. Left ventricular dysfunction along with left atrial dysfunction results in pulmonary venous hypertension and worsening of pulmonary arterial hypertension. This presents clinically in a wide spectrum of labile pre & postductal saturations to profound cyanosis. (*Copyright Satyan Lakshminrusimha)*

Ventricular dysfunction is observed in some patients with severe PPHN due to CDH. During fetal life, the ductus arteriosus serves as a pop-off value and limits right ventricular strain. After birth, remodeled pulmonary vasculature in CDH results in pulmonary hypertension and leads to right ventricle (RV) dysfunction. This is more pronounced after birth when there is excessive strain on the right ventricle. Abnormalities of the left ventricle (LV) have been reported in infants with CDH [26, 40]. When compared to neonates with other causes of PPHN, infants with left sided CDH had significantly lower left ventricular mass assessed by echocardiography. Reduced left ventricular output has been documented in left sided and right sided CDH [41]. The reduced left ventricular mass contributes to functional LV hypoplasia and may result in increased left atrial pressure and pulmonary venous hypertension (Fig. 5) [42].

### Diagnosis

Prenatal diagnosis by ultrasound detects more than 50% of CDH cases at a mean gestational age of 24 weeks [43]. Three-dimensional ultrasound imaging, fetal echocardiography and fetal magnetic resonance imaging (MRI) are other prenatal diagnostic modalities used in assessing the severity and outcome of CDH. Left sided CDH may be characterized by the presence of heterogeneous mass which may be stomach filled with fluid or intestines. In contrast, isolated right-sided CDH is extremely difficult to diagnose by ultrasound if the liver is the only organ that has herniated. Indirect signs such as a shift in cardiac axis, identifying the gall bladder and vasculature in the liver using Doppler may aide in the diagnosis [44]. MRI has been found to be useful in detecting fetal anomalies and can be a valuable adjunct to evaluate the position of the liver and estimating lung volume [45, 46]. Associated cardiac and neural tube defects may affect the outcome of infants with CDH [47].

### Associated syndromes and anomalies requiring genetic work up

Most common associated chromosomal abnormalities are the trisomies 18, 13 and 21 [48]. Chromosomal aneuploidies such as monosomy X, tetrasomy 12 p, tetraploidy 21 have also been associated with CDH [43, 48]. CDH is the most common finding in Fryns syndrome [49]. CDH can also be part of Pentalogy of Cantrell, Apert, Brachmann-Cornelia De Lange, Beckwith-Wiedemann, CHARGE, Coffin-Siris, Goldenhar sequence, Simpson-Golabi-Behmel, Stickler, Pierre Robin sequence and VACTERL [48, 50].

Once diagnosed, the patient should be referred to a tertiary care center for further prenatal workup and management. A multi-disciplinary prenatal consult involving the obstetrics, neonatology, pediatric surgery, genetics at a center that has expertise in managing infants with CDH and extracorporeal membrane oxygenation (ECMO) are imperative. In addition, if an MRI was done, radiology is also involved in the multi-disciplinary prenatal consult.

### Fetal predictors of outcomes

Major determinants of the outcomes in CDH are i) the presence of associated anomalies especially heart disease and ii) extent of lung hypoplasia and (iii) position of the liver [43].

The prognosis of isolated CDH is generally better than CDH complicated by multiple anomalies. Population-based studies report higher survival for isolated CDH compared to CDH with anomalies [4, 51]. Metkus et al. reported higher survival for CDH detected after 25 weeks by ultrasound [52]. This has not been validated and in the true sense, herniation that occurs before 25 weeks tends to have severe lung hypoplasia compared to herniation after 25 weeks [53].

Liver herniation (liver-up) is associated with worse prognosis. Earlier studies have reported 100% survival without liver herniation (liver-down) as compared to 56% with liver herniation [52]. The survival decreased from 74 to 45% with liver herniation as reported by a meta-analysis [54]. In another study, liver herniation was highly predictive of ECMO (80% - liver-up vs. 25% - liver-down) and survival (45% - liver-up vs. 93% liver-down) [55].

**Fig. 6:**
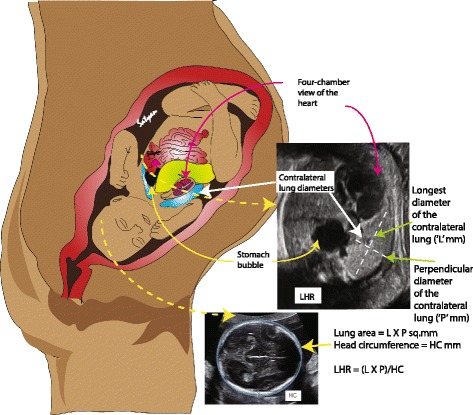
Lung to head ratio (LHR) measurement – Obstetric ultrasound technique is used to measure the lung to head ratio known as LHR to assess the severity of CDH. The head circumference is measured as shown. The contralateral lung area is calculated as a product of the longest and perpendicular diameter of the contralateral lung. The ratio of this area to the head circumference gives the LHR. A LHR of <0.6 has been associated with poor outcome while a ratio of >1.35 has been associated with survival. Alternatively, an observed to expected LHR measurement is used in order to overcome bias secondary to gestational age. *(Copyright Satyan Lakshminrusimha)*

Metkus et al. [52] used the ratio of the contralateral lung size compared with the head circumference to come up with the lung-to-head ratio (LHR, Fig. 6) to assess the severity of pulmonary hypoplasia and to predict postnatal outcome in fetuses with CDH [52, 55, 56]. Since these measurements differed by gestational age and were not found to be consistent across centers [57, 58], observed to expected lung-to–head ratio (O/E LHR) was studied which was independent of gestational age [59]. LHR ratio is often used along with liver herniation to predict outcome (see Table 1 below)

**Table 1:** Antenatal ultrasound predictors of survival in CDH

A. LHR is calculated by dividing fetal lung area (mm^2^) by fetal head circumference (mm). Fetal lung area is usually measured at the level of the four-chamber view of the heart by multiplying the longest diameter of the contralateral lung by its longest perpendicular diameter. Alternatively, some obstetricians trace the lung margin and measure the lung area. The fetal head circumference is measured by its longest electronic ellipse. a. LHR > 1.35 associated with 100% survival b. LHR 1.35 to 0.6 associated with 61% survival c. LHR < 0.6 – no survival
B. Observed to expected LHR (O/E LHR) is calculated by dividing the observed LHR by the expected ratio for gestational age a. The fetal lung area increases 16-fold compared to 4-fold increase in the head circumference between 12 and 32 weeks’ gestation b. O/E LHR < 25% is considered severe CDH (survival 10% with liver up and 25% with liver down) c. O/E LHR < 15% with liver up – 100% mortality
C. Position of liver (or presence of liver herniation) a. Liver herniation with LHR < 1.0 – 60% mortality b. Liver in the thorax – 56% survival;

### Management

#### Antenatal Management – Medical

Antenatal corticosteroids are administered to mothers in some centers to improve lung maturation in neonates with CDH. While some animal results are promising [60], no significant advantages are reported in human infants [61]. It may be prudent to administer antenatal corticosteroids prior to delivery of preterm infants with CDH based on their premature gestation.

Pregnant rats with nitrofen induced CDH demonstrated significant improvement in lung structure, increased pulmonary vessel density, reduced right ventricular hypertrophy following antenatal therapy with high-dose sildenafil [36]. To our knowledge there are no human trials evaluating the role of antenatal phosphodiesterase inhibitors in CDH.

#### Antenatal Management - Surgical

Tracheal occlusion: In the surgically induced lamb model of CDH with hypoplastic lungs, occlusion of the fetal trachea led to an acceleration of lung growth. Harrison et al. at the University of California in San Francisco (UCSF) reported the first randomized controlled trial of open hysterotomy-guided fetal endoscopic tracheal occlusion. No improvement in survival was observed when compared with conventional postnatal care. Junior et al. reported a meta-analysis of various fetoscopic tracheal occlusion studies. Fetoscopic tracheal occlusion procedure increased neonatal survival at 30 days and 6 months among patients with severe CDH. However, it was associated with higher rate of premature rupture of membranes and decreased gestational age at delivery by 2 weeks [62]. A new minimally invasive operation termed percutaneous fetal endoluminal tracheal occlusion (FETO) is being subjected to randomized clinical trials with ongoing recruitment. More information about FETO can be found at http://www.chop.edu/centers-programs/center-fetal-diagnosis-and-treatment/fetoscopic-endoluminal-tracheal-occlusion-feto or http://childrens.memorialhermann.org/FETO-trial/.

#### The timing of delivery

The optimal timing of delivery of an infant with CDH is controversial. Stevens et al. initially reported that among infants delivered by elective cesarean section, early term birth (at 37–38 weeks gestation) was associated with less use of ECMO (22 vs. 35.5%) compared to term delivery (at 39–41 weeks) [63]. However, more recent analysis suggested decreased mortality with advancing gestation [64]. Among 928 infants with CDH in this review, neonatal and infant mortality decreased from 25 and 36% respectively at 37 weeks gestation to 17 and 20% at 40 weeks gestation. We recommend delivery after completion of 39 weeks of gestation to avoid complications associated with prematurity and early term delivery [65].

#### Postnatal Management – Medical (Fig. 7)

##### Delivery room (DR)

Deliveries should be conducted at centers with capabilities of managing an infant with CDH and associated complications. Resuscitation in the DR is based on neonatal resuscitation program (NRP) guidelines [66]. All infants with CDH or suspected CDH need an orogastric/nasogastric tube with suction to decompress the bowel. Bag-mask ventilation should be avoided. The majority of these infants (especially with a prenatal diagnosis of CDH) require intubation in the delivery room. A pre-ductal pulse oximeter is placed on the right upper extremity as soon as possible. Ventilation using a T-piece resuscitator is preferred to avoid high airway pressures. Peak inspiratory pressure (PIP) should be preferably below 25 cm H_2_O to avoid damage to the hypoplastic/immature lung. Oxygen can be titrated to maintain preductal saturations recommended by NRP. In some institutions, preductal saturations > 70% are accepted for the first 1–2 h if pH and arterial carbon dioxide for PaCO_2_ are within normal limits.

**Fig. 7:**
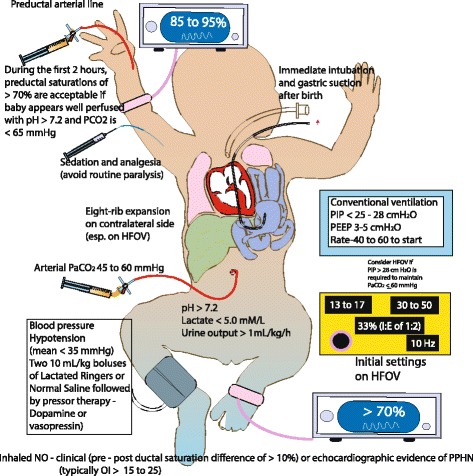
Management of CDH – At birth infants with CDH or suspected CDH should have an orogastric/nasogastric tube with suction to attain bowel decompression. Bag-mask ventilation should be avoided. The majority of these infants (especially with prenatal diagnosis of CDH) require intubation in the delivery room. A pre-ductal pulse oximeter is placed on the right upper extremity as soon as possible. Oxygen saturation targets are based on NRP guidelines. Ventilation using a T-piece resuscitator is preferred to avoid high airway pressures. Ventilator parameters are as shown in the figure. Preductal blood gases and invasive blood pressure monitoring are preferred. Inhaled nitric oxide is often used for the management of PPHN. For blood pressure management, fluid boluses and vasopressor agents are used based on the parameters in the figure. (*Copyright Satyan Lakshminrusimha)*

##### Stabilization

Central or peripheral venous access is obtained for administering fluids and medications. An arterial line for monitoring blood pressure and to draw blood gases is needed. Although it has been traditional to place an umbilical arterial line, it may be preferable to obtain a preductal arterial line in the right radial or ulnar artery. Umbilical artery line values reflect postductal arterial oxygen tension (PaO_2_) and lead to increased fraction of inspired oxygen (FIO_2_). Systemic blood pressures are maintained at normal values for gestational age [67]. Pre-ductal saturations are maintained between 85–95%. A chest x-ray is obtained to assess the initial condition of the lung and herniated content.

##### Mechanical Ventilation

The optimal ventilation mode for infants with CDH and hypoplastic lungs is not known. Many centers initiate conventional mechanical ventilation (CMV) for respiratory support and optimize ventilation by adjusting PIP and respiratory rate. The recently concluded VICI (Ventilation in infants with congenital diaphragmatic hernia) trial compared CMV and high-frequency oscillatory ventilation (HFOV) as the initial mode of ventilation in CDH. There was no statistically significant difference in the combined outcome of mortality or bronchopulmonary dysplasia (BPD) [68]. In this study, 91 (53.2%) patients initially received CMV and 80 (46.8%) HFOV. Forty-one patients (45.1%) randomized to CMV died/had BPD compared with 43 patients (53.8%) in the HFOV group. An odds ratio of 0.62 [95% confidence interval (95% CI) 0.25–1.55] (*P* = 0.31) for death/BPD for CMV vs HFOV was demonstrated, after adjustment for center, LHR, side of the defect, and liver position. Patients initially ventilated by CMV were ventilated for fewer days (*P* = 0.03), less often needed ECMO support (*P* = 0.007), inhaled nitric oxide (iNO, *P* = 0.045), sildenafil (*P* = 0.004), had a shorter duration of vasoactive drugs (*P* = 0.02), and less often failed treatment (*P* = 0.01) as compared with infants initially ventilated by HFOV. It is important to note that guidelines for initial settings for CMV in this study included low positive end-expiratory pressure (PEEP) (3 to 5 cm H_2_O) and PIP (20 to 25 cm H_2_O). These findings suggest that an initial attempt at CMV is reasonable for patients with CDH.

A review of autopsies of 68 infants with CDH showed significant pulmonary injury (alveolar damage, hyaline membrane formation, pneumothoraces in 2/3 of autopsies) secondary to mechanical ventilation where 53 infants were switched to HFOV in a median time of 15 h from birth [69]. In view of preventing volutrauma and barotrauma, a gentler approach to ventilation is preferred in infants with CDH. CMV mode [70, 71] with PIP usually below 25 cm H_2_O and PEEP ≤ 5 cm H_2_O targeting preductal saturations of >85%, post-ductal saturations of >70% and PaCO_2_ of 45–60 mmHg are used to initiate ventilation. Many centers switch to HFOV or jet ventilation as a rescue therapy if the ventilator targets cannot be achieved on CMV. The settings on HFOV are not well defined. Mean airway pressure (MAP) is usually adjusted to maintain adequate inflation of the contralateral lung to 8 ribs in a range of 13–17 cm H_2_O [71–73].

##### The role of surfactant

Although animal studies strongly suggest the presence of an immature lung with surfactant deficiency, a retrospective analysis failed to support any beneficial effect of surfactant replacement therapy in term infants with CDH [74]. Its use in preterm infant was also associated with lower survival rate [75]. However, this trial by its retrospective nature may be biased as sicker patients may have received surfactant Although there are no increasing trends in use of surfactant, it is still being used in preterm infants with CDH across centers [76]. Prospective trials are needed to evaluate the benefits of surfactant in infants with CDH. The beneficial effect of surfactant cannot be ruled out in a premature lung and it is unclear if there is a direct causal association between surfactant administration and mortality in infants with CDH.

##### Hemodynamic monitoring and management

Invasive blood pressure (BP) monitoring is preferred over non-invasive monitoring. Pre and post-ductal saturations and heart rate should be continuously monitored. Optimal end-organ perfusion is the goal to hemodynamic monitoring in infants with CDH. Signs of adequate perfusion include normal range of heart rate for gestational age, normal capillary refill, a urine output >1.0 ml/kg/h, arterial pH >7.2 and lactate levels of <3-5 mmol/L [71]. If there are signs suggestive of poor perfusion, volume resuscitation and vasopressor therapy should be considered. The treatment should be streamlined based on cardiac function assessed by echocardiogram and volume requirement. In case of hypovolemia, a bolus with an isotonic solution like 0.9% normal saline or lactated Ringer’s solution, 10 ml/kg intravenously can be given. Volume resuscitation is usually followed by vasopressor/inotropic therapy.

#### Vasopressor/inotropic therapy (Table 2)

Dopamine is the most commonly used cardiovascular medication in NICU and is given as an infusion [77] aiming to maintain systemic BP appropriate for gestational age. Dobutamine is preferred in infants with poor myocardial contractility. Norepinephrine and epinephrine may be used as first line agents in some institutions secondary to their potent vasoconstrictor activity. Epinephrine infusion can falsely increase the lactate levels and can interfere with management [77]. Low-dose hydrocortisone is beneficial in vasopressor-resistant hypotension in the immediate postnatal period [78]. Vasopressin was reported to be effective in stabilizing systemic hemodynamics in a retrospective chart review with decreased pulmonary/systemic pressure ratio, in patients with CDH [79].

**Table 2:** Vasoactive medications commonly used in CDH: Typical doses, route of administration of inotropic and vasodilator medications in the management of CDH

Drug	Route	Units	Initial dose	Maintenance Dose range
DOPAmine	IV	μg/kg/min	1 to 5	1 to 50 (usually 2.5 to 20)
DOBUTamine	IV	μg/kg/min	1 to 5	1 to 40 (usually 2.5 to 20)
EPInephrine	IV	μg/kg/min	0.05 to 0.1	0.1 to 1
NOREPInephrine	IV	μg/kg/min	0.05 to 0.1	0.05 to 2
PGE1 – Alprostadil	IV	μg/kg/min	0.05 to 0.1	0.01 to 4
PGE1 – Alprostadil	Inhaled	μg/kg/min	0.15 to 0.3	0.15 to 0.3
Milrinone	IV	μg/kg/min	0.25 to 0.75 (some units use a load of 50 μg/kg)	0.25 to 1
Dexamethasone	IV	mg/kg/dose	0.05 to 0.6	0.05 to 0.6
Hydrocortisone	IV	mg/kg/dose	1 to 5	0.5 to 5
Nitric oxide (NO)	Inhaled	Ppm	5 to 20	1 to 80
Vasopressin	IV	Units/kg/min	0.0001 to 0.002	0.0001 to 0.008
Prostacyclin (Epoprostenol - Flolan)	IV	ng/kg/min	1 to 3	50 to 80
Prostacyclin (Epoprostenol - Flolan)	Inhaled	ng/kg/min	50	25 to 50
Prostacyclin (Treprostinil – Remodulin)	SQ or IV	ng/kg/min	1.25 to 2	50 to 80
Prostacyclin (Treprostinil – Remodulin)	Inhaled	μg/breath	6	
Prostacyclin (Iloprost)	Inhaled	μg/breath	2.5 or 5	
Prostacyclin (Beraprost)	PO	μg	80	80 to 120 (adult dose)
Sildenafil	IV	mg/kg/h	0.14 for 3 h	0.07
Bosentan	PO	mg/kg/dose	1 to 2	1 to 2

##### Management of pulmonary hypertension (Fig. 8)

PPHN in CDH infants is secondary to hypoplastic lungs and pruned, remodeled vasculature [39, 80]. Pulmonary arterial hypertension along with left ventricular hypoplasia and right ventricular hypertrophy and/or failure complicated by pulmonary venous hypertension results in severe PPHN not responsive to conventional therapy. Due to right to left shunting, pre and postductal saturation difference may be observed. The absence of a difference does not rule out pulmonary hypertension. In some patients with CDH the immediate postnatal phase, there is a short period of better oxygenation referred to as “honeymoon” period [39]. However, progressive deterioration in oxygenation is commonly observed with deteriorating PPHN. An echocardiogram is the best non-invasive test to assess cardiac function and pulmonary pressures in an infant with CDH and is usually done within the first 24 h and followed up as needed [81].

**Fig. 8:**
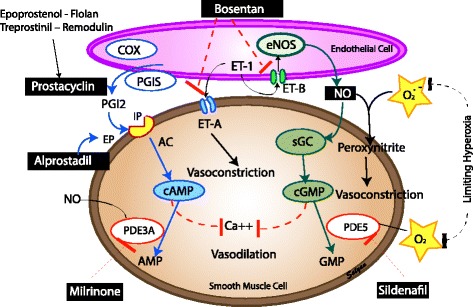
Management of pulmonary hypertension in CDH: pulmonary vasodilators and nitric oxide – prostacyclin – endothelin pathways. AC – acetylcholine, Ca- calcium, cAMP - cyclic adenosine monophosphate, cGMP – cyclic guanosine monophosphate, COX – cycloxygenase, eNOS – endothelial nitric oxide synthase, ET – endothelin, EP – prostaglandin E receptor, IP – prostacyclin I receptor, NO – nitric oxide, PGI – prostaglandin I, sGC - soluble guanylyl cyclase, PDE – phosphodiesterase inhibitor (*Copyright Satyan Lakshminrusimha)*

If preductal saturations decreases below 85%, ventilation adjustments and hemodynamic management take precedence prior to initiating any therapy. Measures to increase systemic blood pressure may minimize the right-to-left shunting. However, there is no need to increase blood pressure to supranormal values if preductal saturation remains above 80%. Catecholamines, especially dopamine, besides increasing systemic vascular resistance also increases pulmonary vascular resistance [30, 82]. The CDH consortium recommends maintaining arterial blood pressure at normal levels for gestational age if preductal saturations are between 80 to 95% [83].

*Inhaled Nitric Oxide (iNO)* is the first agent of choice for treatment of pulmonary hypertension in infants >34 weeks’ gestation. It is a selective pulmonary vasodilator and relaxes pulmonary vascular smooth muscle cells. The criteria for initiating iNO is based on the severity of PPHN as assessed by the oxygenation index (OI). (Note: oxygenation index (OI), = Mean airway pressure x FiO_2_ x 100 ÷ PaO_2_). Oxygen saturation index (OSI) is a non-invasive means of estimating oxygenation status and can be used in the absence of an arterial blood gas but requires further validation [84, 85]. It is calculated using the following formula: Oxygen saturation index (OSI) = Mean airway pressure x FiO_2_ x 100 ÷ preductal SpO_2_). Studies in the past have reported a mean OI of 25±9 as the cut off for initiation of iNO [86]. Currently, in neonates with PPHN not due to CDH, it is acceptable to start iNO with an OI of ≥20 and evidence of right-to-left shunting by clinical exam (a pre - postductal saturation difference of ≥10%) [71] [87] [80] and/or echocardiographic evidence of extrapulmonary right to left shunting [88]. The typical initial dose is 20 parts per million (ppm) [89] although variable dosing has been mentioned in the literature. A complete response to iNO is considered to be an increase in the ratio of arterial oxygen tension (PaO_2_) to fraction of inspired oxygen (FiO_2_) by ≥20 mmHg post iNO therapy [80].

In contrast to PPHN from conditions other than CDH, iNO did not reduce the need for ECMO or death in a prospective, randomized trial in infants with CDH [90]. The ventilation approach, choice of ventilator and the OI at enrollment in this study were different from current practice [33]. In spite of this negative study, iNO continues to be used in US tertiary centers in the management of infants with CDH without a change in ECMO utilization or mortality [91]. If there is no response to iNO after optimizing ventilation and hemodynamic status, iNO is gradually weaned. Some patients decompensate and become hypoxemic with discontinuation of iNO. In these instances, iNO is weaned to a low dose for a few hours and then discontinued.

The rationale for continuing vs. weaning off iNO when there is no response is unclear. Continuing iNO with high oxygen could be detrimental. Nitric oxide is a free radical and can combine with superoxide anions to form peroxynitrite, which is a toxic vasoconstrictor. Thus, continuing iNO therapy in the absence of response remains controversial.

Infants with corrected CDH are at risk for late pulmonary hypertension. Inhaled nitric oxide may play an important role in treating exacerbations of pulmonary hypertension in these patients [88] [92, 93].

*Prostaglandin (PGE1)* intravenous (IV) PGE1 has been used in infants with CDH especially in the setting of right heart failure [94]. A trial of PGE1 to reopen the ductus may reduce the load on the right ventricle. Some groups have suggested starting PGE1 infusion when the duration of right-to-left shunting through the ductus arteriosus was longer than left to right shunting [95]. In patients with ductal-dependent critical congenital heart disease associated with CDH, IV PGE1 is necessary to maintain ductal patency. Inhalational PGE1 is also used as an alternative agent in treating PPHN in infants with CDH [96]. These are non-FDA approved therapies and lack evidence.

*Prostacyclin (PGI*_*2*_ *)* is commonly used in adults may be useful in the management of late pulmonary hypertension in infants post CDH repair. Currently, there is no evidence to support this therapy but some centers use it as a second-line pulmonary vasodilator. Prostacyclin can be used as an inhaled agent or an intravenous agent. Three forms of prostacyclin are used in the management of pulmonary hypertension (Table 2). Epoprostenol (Flolan), Treprostinil (Remodulin) and inhaled Iloprost (Ventavis - inhaled prostacyclin analog) are approved for adults with pulmonary arterial hypertension.

*Sildenafil* is a phosphodiesterase (PDE) 5 inhibitor that inhibits cyclic guanosine monophosphate (cGMP) degradation leading to vasodilation. Oral sildenafil improves oxygenation and reduces mortality in PPHN in centers limited by non-availability of iNO and ECMO [97, 98]. IV sildenafil was shown to be effective in improving oxygenation in patients with PPHN with and without prior exposure to iNO [99]. There are no trials to support its use in infants with CDH. A chronic sildenafil trial was planned in CDH infants but is currently terminated and not recruiting patients (NCT00133679). As per FDA, high mortality is associated with its use in pediatric patients (1–17 y of age) with pulmonary arterial hypertension [100]. Parents should be informed about the benefits and side-effects of sildenafil prior to initiation for chronic use in CDH.

*Milrinone* is a PDE 3 inhibitor which increases cyclic adenosine monophosphate (cAMP) concentration in smooth muscle and myocardium. It has both lusitropic and inotropic properties. In a fetal lamb model of PPHN, milrinone relaxed pulmonary arteries [101] and reduced pulmonary arterial pressure. The benefits of milrinone in children following surgery for congenital heart disease have been well established [102]. Multiple case series have shown IV milrinone to be effective in treating infants with iNO resistant PPHN [103, 104–106]. Milrinone therapy has been used in the management of iNO resistant PPHN in infants with CDH. Hypotension is a clinical concern and the infants should be monitored closely. Despite lack of evidence, the use of milrinone in the management of infants with CDH has increased [76]. A loading dose (50 μg/kg for 30–60 min) followed by a maintenance dose (0.33 μg/kg per minute and escalation to 0.66 and then to 1 μg/kg per minute based on response) are commonly used. The loading dose of milrinone will increase the risk of hypotension but may achieve steady state plasma levels sooner [107]. Hence, the loading dose is not recommended in the presence of systemic hypotension in patients with CDH [108]. Some clinicians administer a volume bolus prior to the loading dose of milrinone to avoid systemic hypotension.

A multicenter trial investigating the use of milrinone in infants with CDH has been proposed by the NICHD Neonatal Research Network and will begin enrollment shortly (NCT02951130).

*Bosentan* is a blocker of endothelin receptors and is occasionally used as an oral agent in the management of chronic pulmonary hypertension in CDH. There is limited experience with its use in neonates [109]. Liver function tests should be closely followed during its use.

*Extracorporeal membranous oxygenation (ECMO)* is considered as the last lifesaving option for infants ≥ 34 weeks’ gestation or with weight >2 kg with CDH and no associated major lethal anomalies after conventional medical management has failed. Strong evidence for ECMO is lacking although the number of infants with CDH who undergo ECMO treatment has not decreased. Selection criteria for ECMO varies across centers and remains controversial. The Euro consortium experts have published criteria [71] for ECMO. There is considerable institutional variability but the following approach seems reasonable – (a) Inability to maintain preductal saturations >85% or postductal saturations >70% along with (b) increased PaCO_2_ and respiratory acidosis with pH <7.15 despite optimal ventilator management, (c) PIP of >28 cm H_2_O or MAP >17 cm H_2_O to achieve saturations >85%, (d) inadequate oxygen delivery with metabolic acidosis, (e) systemic hypotension resistant to fluid and pressor therapy resulting in urine output <0.5 ml/kg/h for a 12–24 h period and (f) consistently elevated OI ≥ 40.

Venoarterial (VA) ECMO is preferred in the presence of cardiovascular compromise. There is a shift towards increased use of venovenous (VV) ECMO in patients with CDH and has comparable mortality rates [110, 111]. Functionally VA ECMO has the advantage of decreasing the load on the right side of the heart. However, VV ECMO allows oxygenated blood to flow through pulmonary circulation resulting in enhanced pulmonary vasodilation while preserving the carotid arteries. In addition, maintaining pulsatile flow with oxygenated blood may improve coronary perfusion and cardiac function with VV ECMO. Failure of VV ECMO and a switch to VA ECMO may be required in some patients.

Duration of treatment on ECMO is a subject of debate. Congenital Diaphragmatic Hernia Study Group retrospective data from 1995–2004 showed increased survival in infants treated for 9 days compared to 14 days [112]. A single institution 19-year retrospective review concluded that in patients with severe CDH, improvement in pulmonary function sufficient to wean from ECMO can take 4 weeks or longer and might need a second ECMO run [113]. The study reported survival rates based on the duration of ECMO run as 56% for 2 weeks, 46% for 3 weeks, 43% for 4 weeks after which the survival dropped to 15%. 44% (7/16) of infants who were treated on the second run of ECMO survived. ELSO registry data [112] shows an increase in mortality for ECMO duration > 2 weeks. Prolonged duration of ECMO is a predictor of mortality [112, 114].

#### Postnatal management - Surgical

When considering repair of the CDH, the surgeon faces three questions: a) what is the benefit; b) when is the optimal time; and c) what is the best approach? The first question, although philosophical, is critical. Our understanding of the benefits of repair are incomplete, but most literature supports the idea that reduction of the herniated visceral contents from the thoracic cavity and closure of the diaphragmatic defect are important in the long-term but provide little immediate benefit to the patient [115]. Reducing herniated contents back to the abdomen to permit expansion of the compressed lungs does not result in immediate improvement in PPHN and hypoxemia. Pulmonary hypertension is, however, the “rate-limiting” disease process in CDH and rarely does hernia reduction/repair significantly improve outcome by itself. This is particularly important as the surgical stress of operation is often severe enough to induce pulmonary hypertensive crises in the sicker patients, and if undertaken in a patient while on ECMO, can lead to severe hemorrhagic complications.

Optimal timing of CDH repair can be difficult to determine, particularly in patients who require ECMO. For those patients not requiring ECMO, repair is usually offered no sooner than 48–72 h after birth, with the assumption that these patients’ pulmonary vasculature is not so compromised as to pose a significant risk of peri- or postoperative decompensation. Once a patient requires ECMO, the decision process becomes more difficult. There are generally three approaches: early repair, immediately after ECMO initiation (typically <72 h); delayed repair, often done as a last-hope operation in the setting of inability to wean off ECMO; and repair after decannulation [116]. The data on the influence of timing of repair on outcomes are hampered by limited numbers, retrospective design, patient group heterogeneity, and thus do not serve well to inform the decision in a generalizable and reliable manner. In broad strokes, best outcomes appear to be in patients repaired after decannulation (technically, these may be repaired the day prior to anticipated decannulation, but in a patient who has improved while on ECMO). Following this, those patients repaired shortly after cannulation do better than those repaired after several weeks on ECMO. Improvements in the medical treatment and prevention of bleeding diatheses while on ECMO, and improved ECMO circuits, have helped to mitigate some of the lethal bleeding complications of repair on ECMO.

### Surgical approach

**Fig. 9:**
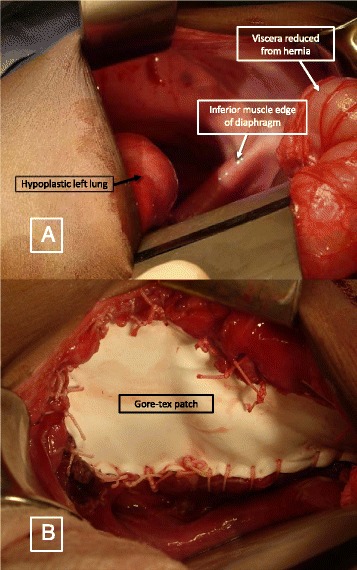
**a** Left sided diaphragmatic hernia showing the hypoplastic left lung, inferior muscle edge of the diaphragm and reduced viscera. **b** Prosthetic patch – Gore-Tex patch used to close the defect

Repair of the CDH may be accomplished through a thoracic or abdominal approach, and may be performed in an open or minimally invasive manner. Long-term outcomes depend, perhaps most importantly, on the characteristics of the diaphragmatic defect. Patients with a small muscular defect that is easily approximated primarily should have negligible recurrence rates and complications. There has been an increasing trend toward thoracoscopic repairs, which are thought to minimize post-operative pain and scarring and hasten recovery. Several series have demonstrated higher recurrences rates through the thoracoscopic approach, although these may have been biased by higher rates of unfavorable anatomy and an inherent learning curve [117]. Patients at the other end of the spectrum, with diaphragmatic agenesis, uniformly require placement of a patch to close the diaphragmatic defect (Fig. 9a & b). These are typically made of synthetic materials (Goretex® is most popular) but there has been growing recent interest in combining synthetic materials with additional biologic layers in an effort to buttress the repair and promote native tissue ingrowth for long-term stability [118]. Lastly, several groups have lauded the benefits of autologous, muscle flap closure of the defect [119].

### Follow-up and outcomes

Infants with CDH face considerable long-term respiratory issues, nutritional problems, neurodevelopmental delays, hernia recurrence and orthopedic deformities [120]. The American Academy of Pediatrics (AAP) came out with guidelines for follow-up of infants who are discharged with CDH [120]. A multidisciplinary approach with long-term follow-up is required for these infants.

Respiratory morbidities include chronic lung disease, rebound pulmonary hypertension, obstructive pulmonary disease and infection. Treatment with ECMO and patch repair were associated with more significant pulmonary morbidity [121] with decreased inspiratory muscle strength. Adolescent survivors often-faced mild to moderate obstructive disease requiring bronchodilator therapy along with weak inspiratory muscle strength [122]. Nutritional problems include gastro esophageal reflux [123, 124] aversion to oral feeds, gastrostomy tube feeding and failure to thrive. Neurological and development problems range from physical disability to neurocognitive and functional delays. Hearing loss is common in these infants [125, 126]. Orthopedic deformities such as pectus and scoliosis are seen in patients post CDH repair [122, 127].

## Conclusion

Despite the unclear etiology of CDH and management of PPHN, over the past few decades, reports have suggested increasing trends of survival in infants with CDH [76]. With the medical and surgical advances in the management of CDH, the reported overall survival is 70-90% [7, 76, 128]. With ECMO, the survival is around 50% [112–114] with different centers reporting different criteria and outcomes. Multiple factors such as prematurity, ECMO, associated abnormalities especially cardiac, need for transport, severity of PPHN, type of repair can affect the outcome and survival of an infant with CDH [51, 114, 128, 129].

## Abbreviations

CDH: Congenital Diaphragmatic hernia
CMV: Conventional mechanical ventilation
ECMO: Extracorporeal Membrane Oxygenation
ELSO: Extracorporeal Life Support Organization
HFOV: High-frequency oscillatory ventilation
iNO: Inhaled nitric oxide
LHR: Lung to head ratio
PPHN: Persistent pulmonary hypertension of newborn
